# Supplementary material for: Multi-scale fusion framework via retinex and transmittance optimization for underwater image enhancement
Source: PLoS One. 2022 Sep 26;17(9):e0275107. doi: 10.1371/journal.pone.0275107 (PMC9512202; doi:10.1371/journal.pone.0275107)
Supplement: Attachment — Submitted filename: PLosOne-Comment.pdf [file pone.0275107.s001.pdf]

<u>Comment</u>

This manuscript presented an underwater image enhancement method based on a fusion of Retinex and transmittance optimization. can deal with turbidity successfully. This method quantified the gray values of each channel to improved the contrast, used the dynamic adaptive stretch compensation to solve the color deviation, and obtained the restored image through an inverse double transmittance algorithm. Multi-scale pixel-level fusion is used to construct the Laplacian and Gaussian pyramids for input and weight maps, resulting in the final underwater image.

The novelty of this manuscript is limited in an integration and tinkering of existing methods, and the runtime of the proposed method may be disadvantageous in practical applications. Practically speaking, the innovation of the whole paper has not reached the level of this journal. You can't write a paper for the sake of delivering it, but really solve practical problems.

Some comments should be concerned below:
C1. The proposed method should be proved in some real videos of underwater scenes.
C2. The runtime of the proposed method should be compared with some non deep learning and deep learning state-of-the-art methods on some public datasets.
C3. The mainline of Introduction should be clear so that readers can better understand their intentions.
C4. There are some formatting problems, such as basic alignment of all paragraphs.
C5. There is an error in Fig. 1 where the reflected light direction of suspended particles is not uniform.

Missing references:

[1] Underwater Image Enhancement via Medium Transmission-Guided Multi-Color Space Embedding. Chongyi Li, Saeed Anwar, Junhui Hou, Runmin Cong, Chunle Guo, Wenqi Ren. IEEE Transactions on Image Processing, 2021.

[2] Bayesian Retinex Underwater Image Enhancement. Peixian Zhuang, Chongyi Li, Jiamin Wu. Engineering Applications of Artificial Intelligence, 2021.
