# Supplementary material for: Multi-scale fusion framework via retinex and transmittance optimization for underwater image enhancement
Source: PLoS One. 2022 Sep 26;17(9):e0275107. doi: 10.1371/journal.pone.0275107 (PMC9512202; doi:10.1371/journal.pone.0275107)
Supplement: Attachment — Submitted filename: Response to Reviewers.pdf [file pone.0275107.s003.pdf]

Response to Reviewers

To Reviewer #1:

Question #1:

The proposed method should be proved in some real videos of underwater scenes.

Author response:

Thank you for your questions about the practical application of our real video in underwater scenes. Your questions are indeed valuable for us to perfect our paper. We supplemented the comparison problem in the application test in sub-section 3.6 of the manuscript. The real underwater video and enhanced video have been uploaded to Google's online disk. The modified content of the text is as follows:

Finally, in order to reflect the practical utilization of our work, our method is applied to underwater real video. The original video is decomposed into one frame at a frame rate of 30 frames per second, then each frame is fused and enhanced, and the corrected video is finally synthesized. As shown in Figure 14, two original images of three underwater videos are intercepted for experimental comparison. The real underwater video and enhanced video have been uploaded to Google Drive.

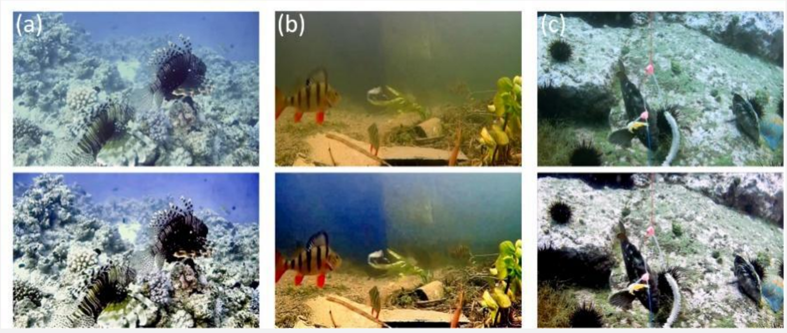

Fig 14. Real underwater video test. (a) Image from video 1; (b) Image from video 2; (c) Image from video 3.

The experimental results show that the pretreatment effect of underwater video is clearer, and can reflect more abundant environmental information, which has practical application value.

Question #2:

The runtime of the proposed method should be compared with some non-deep learning and deep learning state-of-the-art methods on some public datasets.

Author response: We are so grateful for the recommended evaluation factor, running speed. The running speeds of non-deep learning and deep learning algorithms have been evaluated and shown in Table 5 in sub-section 3.4. Among them, non-deep learning algorithms include UDCP, DRDCP, Fusion and UNTV. Deep learning algorithms include MLFcGAN, FUnIEGAN, UWCNN and WaterNet. The modified content of the text is as follows:

We compare the average time this code runs on three common datasets with non-deep learning and deep learning methods to analyze the complexity of the algorithm (CPU: Intel i7-6700HQ 2.60GHz;

GPU: NVIDIA RTX 2070 8GB). Among them, non-deep learning algorithms include UDCP, DRDCP, Fusion, and UNTV. Deep learning algorithms include MLFcGAN, FUnIEGAN, UWCNN, and WaterNet. The running time of deep learning is only the test time, excluding the training time. The average processing time of each method is shown in Table 5.

Table 5. Average running time (seconds).

Methods	UDCP	DRDCP	Fusion	UNTV	MLFc-GAN	FUnIE-GAN	UWCNN	Water-Net	Ours
Average	0.181	0.237	1.172	2.428	1.424	1.826	1.701	8.301	1.351

We note that our method runs slower than the UDCP and DRDCP when only considering the non-deep learning methods. The reason is that we deal with the contrast, color, and dehazing of the underwater image separately, and the additional image enhancement effect increases the runtime. However, the running speed of ours is better than that of Fusion and UNTV, which basically meets the requirements of underwater real-time performance. For deep learning algorithms, not only takes one to two days to train, but also consumes a lot of memory, and the test time is inferior to traditional algorithms. According to various subjective and objective evaluation metrics, our method outperforms other single restoration methods when dealing with complex underwater environments. Therefore, our work still has strong advantages in terms of comprehensive speed and enhancement effect.

Question #3:

The mainline of Introduction should be clear so that readers can better understand their intentions.

Author response: Thank you very much for your suggestions on the structure and mainlines of the Introduction. Your questions are indeed valuable for us to perfect our paper. As suggested by the reviewers, we have divided the Introduction into four parts to make it more logical. Firstly, the research status is drawn from the research background and significance. Current underwater image processing includes enhancement, restoration, and deep learning. In order to make the mainline of the Introduction clearer, we adjusted the order of the existing literature and introduced the representative works of the three types of methods according to the progress of time. Then, we expound on the necessity and innovation of this work. Finally, the chapter arrangement of this paper is briefly introduced. The modified content of the text is as follows:

In order to improve the detailed information of underwater images, researchers have proposed various image sharpening methods as preprocessing techniques to restore explicit and natural underwater scenes, including underwater image enhancement, restoration based on physical models and deep learning. Next, we introduce the representative works of the three types of methods according to the progress of time.

The image pixels are processed directly in most underwater image enhancement algorithms. High-contrast images usually exhibit the characteristics of rich details and an extended dynamic range. Contrast limited adaptive histogram equalization (CLAHE) [1] algorithm can improve the contrast of underwater images, but it will introduce noise. The purpose of Retinex theory is to remove the influence of illumination light from an image to obtain the reflection attribute of an object. Yang et

al. [2] decomposed V-channel via wavelet transform; then employed denoising algorithm with soft threshold and locally adaptive tone mapping to address the high-frequency and low-frequency components, respectively. Hu et al. [3] further improved its parameters and optimized the performance based on the classical multi-scale Retinex (MSR). Zhang et al. [4] applied a Gaussian low-pass filter to the L channel. They processed low-frequency components through optimal equalization threshold strategy of double interval histogram and enhanced high-frequency components with S-shaped function. Alternatively, Huang et al. [5] used the power function enhancement algorithm for visible light to improve the contrast, corrected the brightness of infrared image, and fused the two versions with Laplace transform. Zhuang et al. [6] established a posterior formulation for underwater image enhancement by imposing multi-step gradient priors on reflectance and illuminance. A piecewise and piecewise linear approximation of reflectivity is modeled with the $l1$ norm, and the $l2$ norm is used to enhance spatial smoothness and spatial linear smoothness over lighting. Clear images are obtained through convergence analysis and optimization.

Jaffe-McGlamery's underwater optical imaging model [7] has been widely applied in underwater image restoration algorithms, shown in Figure 1. Among them, He et al. [8] proposed the dark channel prior (DCP) theory built upon extensive statistical research, which indicates that there is at least one low-intensity channel in the red, green, and blue color channels in fog-free images. Consequently, the dark channel prior theory is widely used to estimate the background light and transmittance of haze images. However, since the attenuation characteristics of underwater light are different from that of the land environment, this algorithm cannot get superior results when it is directly applied to underwater images. Based on the DCP, Peng et al. [9] combined image blurriness and red light absorption differences to estimate the scene depth map and estimate the background light from the blurred area. This method is more suitable for underwater scenes. Drew et al. [10] proposed a priori method of underwater dark channels, which obtained relatively accurate transmittance based on the assumption that most visual information was heavily altered in the blue and green channels. Emberton et al. [11] divided the input image into three categories (bluish, greenish, and bluish-green) and restored it according to the color deviation characteristics of each region. Besides, Chang et al. [12] revised the SDCP scheme to make DCP suitable for underwater environments, and adopted point spread function deconvolution strategy to solve the blurred edges.

With the vigorous development of networks, deep learning has been gradually applied to image processing in recent years. Jamadandi et al. [13] utilized the VGG19 network as the encoder and mirrored the wavelet non-pooling layer as the decoder to reconstruct the image model. Lin et al. [14] adopted the deep learning and rectified linear unit (ReLU) activation function to compensate for the linear model through the addition of nonlinear factors and effective feature achievement. Islam et al. [15] proposed FUnIE-GAN based on the U-Net framework. In order to obtain rich feature information, residual connections are added to the generator. To reduce the semantic difference between low-level features and high-level features, Han et al. [16] added residual path blocks between encoders and adopted depth supervision mechanism to improve gradient propagation. Lin et al. [17] proposed a multi-scale deformable convolutional network composed of encoder-decoder, which acquires abundant feature information of the receptive field from different scales, and finally optimized the model through pixel loss and perceptual loss. Li et al. [18] introduced a physical model on the basis of deep learning and designed a medium-transport guided decoder network to enhance

the network's response to quality-degraded regions.

Question #4:

There are some formatting problems, such as basic alignment of all paragraphs.

Author response: Thank you for pointing out the formatting errors in our manuscript. We all agree with this comment, and we feel very sorry for the existence of the errors. The manuscript has been carefully checked and edited to improve formatting.

Question #5:

There is an error in Fig. 1 where the reflected light direction of suspended particles is not uniform.

Author response: Thanks for pointing out the bug in Fig. 1. We have modified the direction of the reflected light from the suspended particles in Fig.1 to ensure that the image conforms to the physics. In addition, we checked all illustrations for format and correctness, making them visible in the revised manuscript. Fig.1 is modified as follows:

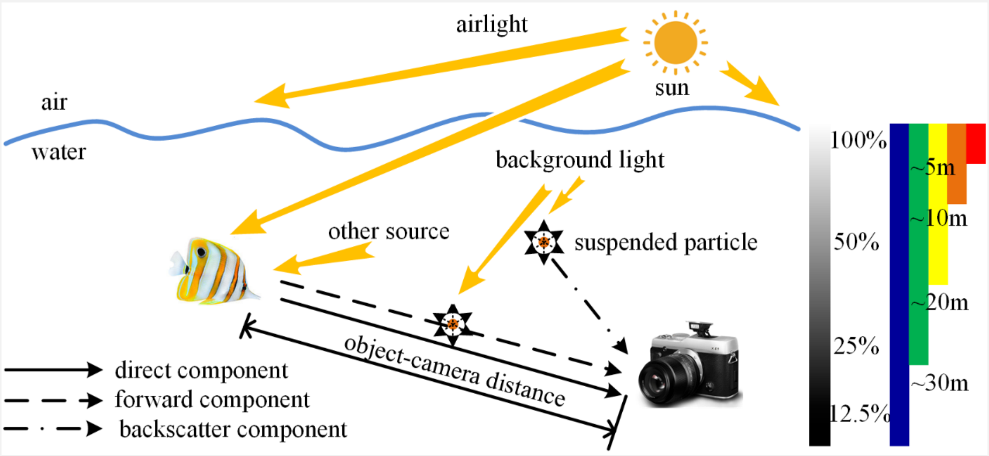

Fig 1. Underwater optical imaging model.

Question #6:

Missing references:

[1] Underwater Image Enhancement via Medium Transmission-Guided Multi-Color Space Embedding. Chongyi Li, Saeed Anwar, Junhui Hou, Runmin Cong, Chunle Guo, Wenqi Ren. IEEE Transactions on Image Processing, 2021.

[2] Bayesian Retinex Underwater Image Enhancement. Peixian Zhuang, Chongyi Li, Jiamin Wu. Engineering Applications of Artificial Intelligence, 2021.

Author response: We are so grateful for your suggestion on the references. According to the surveys in the recommended literature [1] and [2], we modified the Introduction section and made it readable. The modified content of the text is as follows:

Zhuang et al. [6] established a posterior formulation for underwater image enhancement by imposing

multi-step gradient priors on reflectance and illuminance. A piecewise and piecewise linear approximation of reflectivity is modeled with the l_1 norm, and the l_2 norm is used to enhance spatial smoothness and spatial linear smoothness over lighting. Clear images are obtained through convergence analysis and optimization.

Li et al. [18] introduced a physical model on the basis of deep learning and designed a medium-transport guided decoder network to enhance the network's response to quality-degraded regions.

To Reviewer #2:

Question #1:

most of the methods included in the overall framework of this method are already well-known. In the proposed methodology, some integrated methods are combined in sequence to get better human perception results. In this way, the manuscript holds novelty. However, the author must discuss their original contribution.

Author response: We greatly appreciate your suggestions for our original contribution. Your questions are indeed valuable for us to perfect our paper. Therefore, to give readers a clearer understanding of our intentions, we have modified the contribution in the Methodology section to illustrate the effectiveness of the fusion algorithm in our proposed method. The modified content of the text is as follows:

Generally speaking, our main contributions are summarized as follows. (1) We design three images I, II, and III respectively as the input of the fusion framework. I. Histogram quantization is performed on the middle gray area of each channel to improve contrast. II. Image 2 adopts dynamic adaptive compensation to replace the linear transformation in the color constancy MSR algorithm to correct the image color. III. Innovatively applies the dual transmittance imaging model to the underwater image fusion framework and integrates the red channel prior to the total transmittance estimation, which complements the transmittance information and removes the image turbidity. (2) Proposed method extracts the weights of the preprocessed clear input images. After obtaining features and necessary information of the same scene and target, various algorithms are fused to emphasize the image details. (3) Comprehensive and particular verification of the advantages of the algorithm through color correction testing, qualitative and quantitative comparison, complexity analysis, ablation experiments, and application analysis in real underwater. As shown in Figure 2, our study modifies and improves traditional algorithms, resulting in a comprehensive increase in performance for underwater image enhancement.

Question #2:

In section 2 Methodology, improved multi-scale Retinex with color preservation (IMSRCP), histogram quantization, the red channel prior is integrated to the total transmittance estimation, and extracting the weights of the preprocessed clear input images. But in the explanation of methodology in each sub-sections, the order of the methodology seems to be in correct. Reorder the methods used in the methodology based on the summary as briefed in the overview of the proposed methodology of Section 2.

Author response: Thank you for your question about the ordering of our methods in Section 2. We are very sorry that the previous work was not careful enough. Your suggestions are valuable in improving the visibility of our paper. We have reordered the overview of the proposed method in Section 2 according to the arrangement of the chapters. And the full text of the abstract, Figure 2, conclusion, and so on a comprehensive check. The modified content of the text is as follows:

We design three images I, II, and III respectively as the input of the fusion framework. I. Histogram

quantization is performed on the middle gray area of each channel to improve contrast. II. Image 2 adopts dynamic adaptive compensation to replace the linear transformation in the color constancy MSR algorithm to correct the image color. III. Innovatively applies the dual transmittance imaging model to the underwater image fusion framework and integrates the red channel prior to the total transmittance estimation, which complements the transmittance information and removes the image turbidity.

Question #3:

In sub-section 2.2 Design weight diagrams, the normalized weight map of the k-th input image, K = 3. But in the Fig. 2, the decomposition level is 5. Explanations are needed because this introduces ambiguity.

Author response: Thanks for your comments. We are very sorry for the misunderstanding caused by our unclear statement. In this paper, we design three input images that include enhanced contrast, color correction, and removing atomization. The total number of types of input images is K=3 in sub-section 2.2. For the input images, we also obtained three weight maps for each input map, including Luminance weight map, chromatic weight map, and saliency weight map. Then, the three weight maps of each input image are normalized to obtain the standardized weight map. Finally, we decompose each of the three input images into a five-layer Laplacian pyramid and each of the corresponding three normalized images into a five-layer Gaussian pyramid. The five-level decomposition level in Figure 2 is the number of pyramid levels in sub-section 2.3. In order to simplify Figure 2, we only show the de-atomized weight map and pyramid level decomposition in the figure. We have revised Figure 2 and its annotations to address the confusion this paper brings to readers. In sub-section 2.3, in order to distinguish it from L in sub-section 2.2, the symbol n of the pyramid level is redefined and the total number of pyramid levels N=5 is indicated. The modified content of the text is as follows:

$$R_n(x) = \sum_{k=1}^{K} G_n\{\bar{W}_k(x)\} LP_n\{I_k(x)\} \tag{18}$$

where n and k determine the number of pyramid layers and input images, in this paper, the total number of layers of the pyramid is N=5, Gn represents the n layer of Gaussian decomposition of the normalized weight map and LPn represents the n layer of Laplace decomposition.

Question #4:

the Proposed Methodology Fig. 2 needs a self-explanation with proper order of the methods used, and further, the Fig. 2 needs more artistic representation and check for the normalized weight map and its corresponding level of decomposition.

Author response: Thank you for your suggestions on the annotation and layout of Fig. 2. Your suggestions are very helpful to improve our paper. We have added relevant sequential explanations of the methods used in the annotations to Fig. 2, retyped Fig. 2 to make it more artistic, and updated the normalized weight map and its corresponding level of decomposition. Fig.2 is modified as follows:

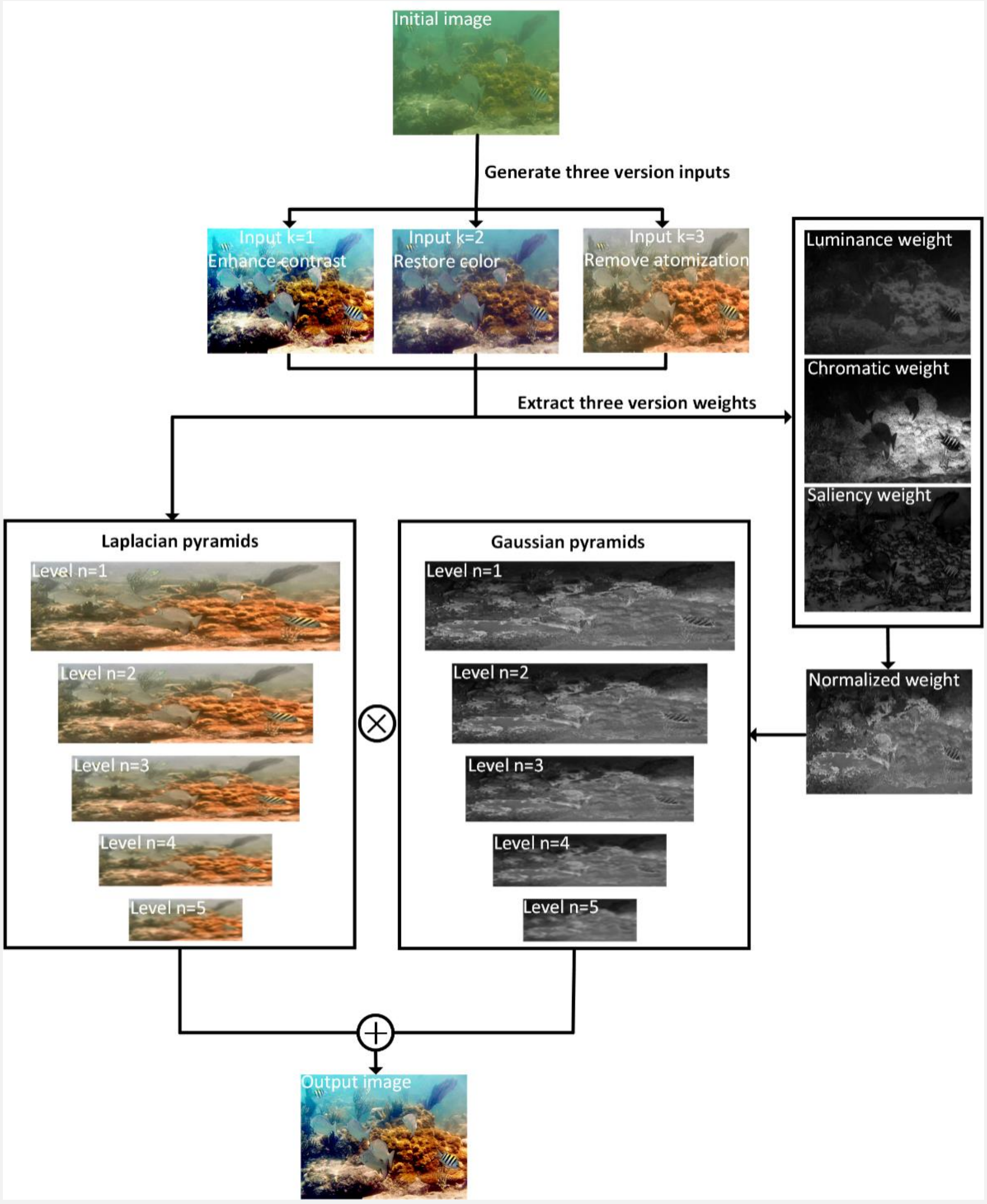

Fig 2. The three input images including contrast enhancement, color correction, and defogging are decomposed into five-layer Laplacian pyramids, respectively, and its normalized weight map is decomposed into five-layer Gaussian pyramids. (To simplify the flowchart, we only show the defogging weight map and its pyramid level decomposition in the figure.) Finally, multi-scale fusion is performed to obtain the output image.

Question #5:

Some ablation studies need to be performed in the experiments to prove the effectiveness of each method. If possible PSNR validation can be carried out in ablation study.

Author response: Thank you for your questions about the existential value of each component in the paper. Your questions are valuable for us to validate the necessity of each module. We have performed ablation experiments and compared them with the original algorithm using the PSNR

evaluation metric. Additional ablation studies are as follows:

In this sub-section, we verify that each input component is indispensable with the PSNR evaluation metric. A denotes ours without contrast enhancement component, B represents ours without color correction component, C indicates ours without defogging component, and D is the complete frame. The PSNR index is a full reference evaluation, which needs to be compared with the ground truth. Images with numerically higher PSNR values are generally considered better quality. However, there are no ground truth images in the RUIE dataset, so only the mean PSNR values on the UIEBD and EUVP datasets are compared, as shown in Figure 11, the red font is the optimal value. We note that the PSNR values of the unremoved input modules are all higher than other models, indicating that the image restoration effect is the best, close to the natural state.

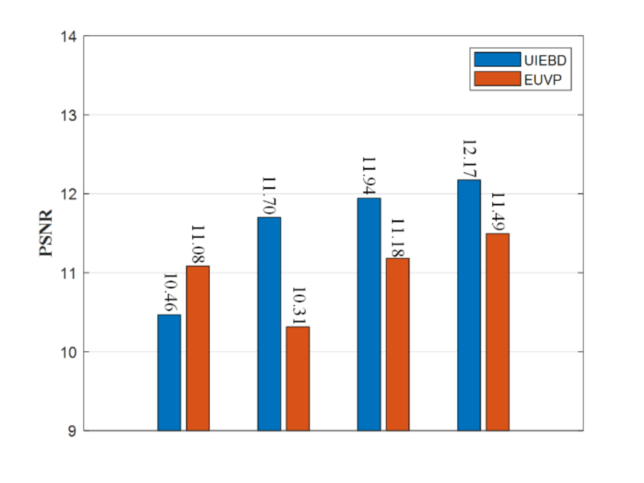

Fig 11. Comparison of ablation studies

Question #6:

the author must discuss failure cases of their method (if any).

Author response: Thank you for the reviewer's question of the failure cases. Your doubt is valuable for us to improve our paper. We have included the limitations of our proposed method in the discussion.

Our method generally outperforms other contrasting algorithms, but still has certain limitations. For example, in UCIQE and UIQM indicators, the algorithm is not the optimal value. The background regions of the deeply blurred states in image 8 and image 10 in Figure 9 cannot be effectively recovered. In the processing of real underwater video, it is found that it cannot process low-resolution moving images. At the same time, according to the comparative running time, the complexity of the algorithm needs to be further improved.

Question #7:

there are few typo errors present in the manuscript, the author must correct these errors.

Author response: We are so grateful for your careful review. For sure, the English expression and accurate description are essential to scientific papers. Following the advice of typos, we have checked and polished the whole paper carefully.

Question #8:

notations used in certain equations were not properly defined.

Author response: Thanks for your suggestion about the notation in the equations, we are very sorry for these errors. Your suggestion is very helpful for us to improve the correctness of the paper. After careful review and checking, we have made changes to the symbols in the following equations.

(1) In order to unify the R, G, and B color channels in the text, we change i in equation (3) and equation (4) to c, $c \in \{R, G, B\}$, which is consistent with equation (5).

$$\begin{cases} I^c_{\text{msr\_min}} = I^c_{\text{msr\_mean}} - \mu I^c_{\text{msr\_var}} \\ I^c_{\text{msr\_max}} = I^c_{\text{msr\_mean}} + \mu I^c_{\text{msr\_var}} \\ I^c_{\text{imsr}} = \dfrac{R^c_{\text{msr}} - I^c_{\text{min}}}{I^c_{\text{max}} - I^c_{\text{min}}} \times 255 \end{cases} \tag{3}$$

$$I^c_{\text{imsrcp}} = \rho \frac{I^c_{\text{mean}}}{I^L_{\text{imsr\_mean}}} I^c_{\text{imsr}} \tag{4}$$

$$I_c(x) = J_c(x) t_{\text{D}}(x) + \left[1 - t_{\text{B}}(x)\right] \cdot A_c \tag{5}$$

(2) In order to keep the chromatic map W_E consistent with the luminance map W_L and saliency map W_S format in equation (14), we change the notation of the chromatic map to W_C.

$$W_C = \exp\left(-\frac{\left(S(x) - S_{\max}\right)^2}{2\sigma^2} \right) \tag{14}$$

$$W_k = W^k_{\text{L}} + W^k_C + W^k_{\text{S}} \tag{16}$$

(3) In Equation (18), in order to distinguish it from the average value L of three-channel pixels in Equation (13), we modify the number of pyramid layers l to n.

$$R_n(x) = \sum_{k=1}^{K} G_n\{\bar{W}_k(x)\} LP_n\{I_k(x)\} \tag{18}$$

where n and k determine the number of pyramid layers and input images, in this paper, the total number of layers of the pyramid is N=5, G_n represents the n layer of Gaussian decomposition of the normalized weight map and LP_n represents the n layer of Laplace decomposition.

Question #9:

the english of the paper must be revised thoroughly as the reviewer has found numerous grammatical errors and punctuation mistakes.

Author response: Thank you for pointing out the errors in our manuscript and the problem with our grammar and punctuation. We all agree with this comment, and we feel very sorry for the existence of the errors. The manuscript has been carefully edited to eliminate typos and improve the language and format.

Question #10:

the work is very interesting, however too many algorithms will lead to consume more time and computing power.

Author response: Thank you very much for questioning the complexity of the algorithm, just like the first reviewer. Your comments are very valuable for perfecting our thesis. Therefore, we compare the running time of each algorithm, shown in Table 5 in sub-section 3.4.

We compare the average time this code runs on three common datasets with non-deep learning and deep learning methods to analyze the complexity of the algorithm (CPU: Intel i7-6700HQ 2.60GHz; GPU: NVIDIA RTX 2070 8GB). Among them, non-deep learning algorithms include UDCP, DRDCP, Fusion, and UNTV. Deep learning algorithms include MLFcGAN, FUnIEGAN, UWCNN, and WaterNet. The running time of deep learning is only the test time, excluding the training time. The average processing time of each method is shown in Table 5.

Table 5. Average running time (seconds).

Methods	UDCP	DRDCP	Fusion	UNTV	MLFc-GAN	FUnIE-GAN	UWCNN	Water-Net	Ours
Average	0.181	0.237	1.172	2.428	1.424	1.826	1.701	8.301	1.351

We note that our method runs slower than the UDCP and DRDCP when only considering the non-deep learning methods. The reason is that we deal with the contrast, color, and dehazing of the underwater image separately, and the additional image enhancement effect increases the runtime. However, the running speed of ours is better than that of Fusion and UNTV, which basically meets the requirements of underwater real-time performance. For deep learning algorithms, it not only takes one to two days to train, but also consumes a lot of memory, and the test time is inferior to traditional algorithms. According to various subjective and objective evaluation metrics, our method outperforms other single restoration methods when dealing with complex underwater environments. Therefore, our work still has strong advantages in terms of comprehensive speed and enhancement effect.
