# Supplementary material for: Multi-scale fusion framework via retinex and transmittance optimization for underwater image enhancement
Source: PLoS One. 2022 Sep 26;17(9):e0275107. doi: 10.1371/journal.pone.0275107 (PMC9512202; doi:10.1371/journal.pone.0275107)
Supplement: Attachment — Submitted filename: Response to Reviewers.pdf [file pone.0275107.s005.pdf]

Response to Reviewers

To Reviewer #1:

Question #1:

We have no comments on this manuscript, and recommend the authors to check the full text carefully. This manuscript presented an underwater image enhancement method based on a fusion of Retinex and transmittance optimization. This method quantified the gray values of each channel to improved the contrast, used the dynamic adaptive stretch compensation to solve the color deviation, and obtained the restored image through an inverse double transmittance algorithm. Multi-scale pixel-level fusion is used to construct the Laplacian and Gaussian pyramids for input and weight maps, resulting in the final underwater image.

Author response:

Thank you for giving us the opportunity to improve our manuscript. Your comments are positive and constructive. Your review has improved the academic level and publication quality of the article. Thank you again for your recognition and affirmation of our manuscript. Having said this, we remain open to any additional suggestions that you might subsequently offer. Additionally, should it be that we either did not fully address a point or misinterpreted a point we remain more than willing to make subsequent alterations to the manuscript.

To Reviewer #2:

Question #1:

The paper title should be concise and as short as possible, and include keywords(refer - as per Authors Guidelines of this journal).

Author response:

Thank you for your professional suggestions on the title of our paper. We have referred to the journal author's guide and revised the title of our paper as required. The revised thesis title is as follows:

Multi-scale fusion framework via retinex and transmittance optimization for underwater image enhancement.

Question #2:

I appreciate that the authors has carried out the Ablation study based on PSNR metric and why not other metrics such as UCIQE and UIQM metrics. Further, the authors can also include Ablation study with input image and its corresponding output. This can provide the readers to understand the variations in the output image as well as the importance of each block of components as proposed in Fig 2.

Author response:

Thank you for your suggestions on the lack of UCIQE and UIQM indicators and the importance of each module in the ablation experiment in the paper. Your suggestions are of great value to improve our paper. Therefore, we added UCIQE and UIQM evaluation indicators in ablation experiments, and analyzed the output of each module to further verify the effectiveness of the algorithm. The amendment is as follows:

In this sub-section, the input image of each module component is compared with the fused clear image for detail information recovery experiment, as shown in Fig 11, the upper right corner is the local red mark area. The analysis shows that the input image can effectively complete contrast enhancement, color correction and dehazing respectively. Excessive color correction of the second input image will lead to serious exposure. The third input image can eliminate dense haze, but with distorted brightness and color. In contrast, our method establishes more generality than three input images that lack optimization capabilities.

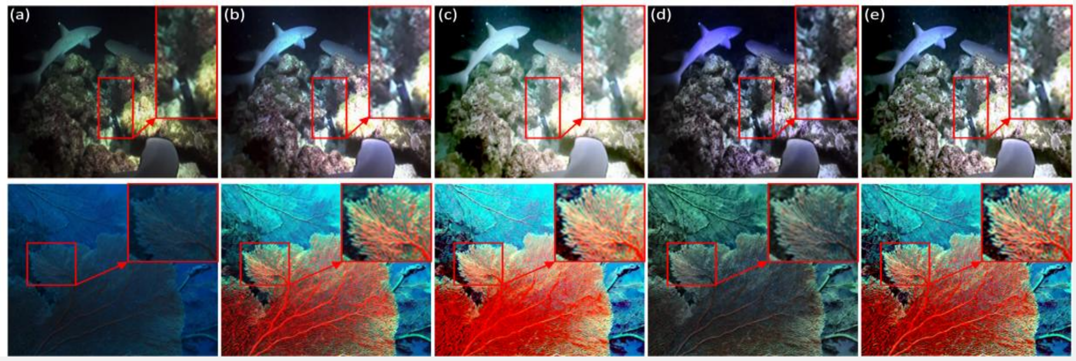

Fig 11. Comparison of details of each input component. (a) Degraded image; (b) First input image; (c) Second input image; (d) Third input image; (e) Ours.

After that, we verify that each input component is indispensable to the PSNR, UCIQE and UIQM evaluation metric. A denotes ours without contrast enhancement component, B represents ours without color correction component, C indicates ours without defogging component and D is the complete frame. The PSNR index is a full reference evaluation, which needs to be compared with the ground truth. Images with numerically higher PSNR values are generally considered better quality. However, there are no ground truth images in the RUIE dataset, so only the mean PSNR values on the UIEBD and EUVP datasets are compared. UCIQE and UIQM are non reference evaluation indicators, we compared the values of each component on the three data sets, as shown in Fig 12. We note that the PSNR, UCIQE and UIQM values of the unremoved input modules are all higher than other models, indicating that each module of input plays an important role.

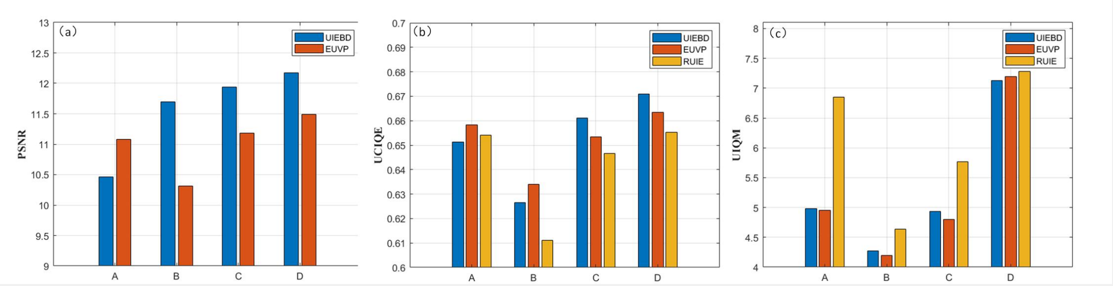

Fig 13. PSNR, UCIQE and UIQM values of ablation experiment. (a)PSNR, (b) UCIQE, (c) UIQM.

Question #3:

Make sure all figures are as per the journal dpi format, since some of the images are seems to be of less resolution (refer - as per Authors Guidelines of this journal).

Author response:

Thank you for your suggestions, which are very helpful to improve our paper format. We have carefully checked whether the resolution of each figure meets the requirements of the journal, and revised it according to the standard. When uploading figures, the system automatically generates PDF that reduces the resolution, resulting in the degradation of uploaded figures quality. Readers can click the link in the upper right corner of each figure to download the original figure to improve the visual effect.

Question #4:

English grammar correction (such as punctuation, typo mistakes) needs to be done.

Thank you for pointing out the errors in our manuscript and the problem with our punctuation and typo. We all agree with this comment, and we feel very sorry for the existence of the errors. The manuscript has been carefully edited to eliminate typos and improve the language and format.
