# Supplementary material for: Multi-scale fusion framework via retinex and transmittance optimization for underwater image enhancement
Source: PLoS One. 2022 Sep 26;17(9):e0275107. doi: 10.1371/journal.pone.0275107 (PMC9512202; doi:10.1371/journal.pone.0275107)
Supplement: Attachment — Submitted filename: PONE-D-22-07396 Reviewer comments.docx [file pone.0275107.s002.docx]

The paper consists of a series of image enhancement algorithms such as improved multi-scale Retinex with color preservation (IMSRCP), histogram quantization, the red channel prior is integrated to the total transmittance estimation, and extracting the

weights of the preprocessed clear input images. However, I have a few questions:

-- most of the methods included in the overall framework of this method are already well-known. In the proposed methodology, some integrated methods are combined in sequence to get better human perception results. In this way, the manuscript holds novelty. However, the author must discuss their original contribution.

-- In section 2 Methodology, improved multi-scale Retinex with color preservation (IMSRCP), histogram quantization, the red channel prior is integrated to the total transmittance estimation, and extracting the weights of the preprocessed clear input images. But in the explanation of methodology in each sub-sections, the order of the methodology seems to be in correct. Reorder the methods used in the methodology based on the summary as briefed in the overview of the proposed methodology of Section 2.

-- In sub-section 2.2 Design weight diagrams, the normalized weight map of

the k-th input image, K = 3. But in the Fig. 2, the decomposition level is 5. Explanations are needed because this introduces ambiguity.

-- the Proposed Methodology Fig. 2 needs a self-explanation with proper order of the methods used, and further, the Fig. 2 needs more artistic representation and check for the normalized weight map and its corresponding level of decomposition.

-- Some ablation studies need to be performed in the experiments to prove the effectiveness of each method. If possible PSNR validation can be carried out in ablation study.

-- the author must discuss failure cases of their method (if any).

-- there are few typo errors present in the manuscript, the author must correct these errors.

-- notations used in certain equations were not properly defined.

-- the english of the paper must be revised thoroughly as the reviewer has found numerous grammatical errors and punctuation mistakes.

-- the work is very interesting, however too many algorithms will lead to consume more time and computing power.
