# Supplementary material for: Multi-scale fusion framework via retinex and transmittance optimization for underwater image enhancement
Source: PLoS One. 2022 Sep 26;17(9):e0275107. doi: 10.1371/journal.pone.0275107 (PMC9512202; doi:10.1371/journal.pone.0275107)
Supplement: Attachment — Submitted filename: Reviewer Comments.docx [file pone.0275107.s004.docx]

Reviewer Comments:

1. The paper's title should be concise and as short as possible, and Include 10 keywords (refer - as per Authors Guidelines of this journal)
2. I appreciate that the authors has carried out the Ablation study, only for PSNR has been carried out and why not other metrics such as UCIQE and UIQM metrics. Further, the authors can include Ablation study with input image and its corresponding output. This can provide the readers to understand the variations in output image and importance of each block of components as proposed in Fig. 2.
3. Make sure all figures are as per the journal dpi format, since some of the images are seems to be of less resolution (refer - as per Authors Guidelines of this journal).
4. English grammar correction (such as punctuation, typo mistakes) needs to be done.
